# Clinical presentation, management and outcomes of bile duct injuries after laparoscopic cholecystectomy: a 15-year single-center experience in Vietnam

**DOI:** 10.3389/fsurg.2023.1280383

**Published:** 2023-10-11

**Authors:** Hung Quang Vu, Duc Trong Quach, Bac Hoang Nguyen, Anh-Tuan Quan Le, Nhan Quang Le, Hai Minh Pham, Ngoc-Huy Thai Tran, Dang-Khoa Hang Nguyen, Ngoc-Sang Thi Duong, Toan Van Tran, Binh Long Pham

**Affiliations:** ^1^ Department of General Surgery, University of Medicine and Pharmacy at Ho Chi Minh City, Ho Chi Minh City, Vietnam; ^2^ Department of Hepatobiliary and Pancreatic Surgery, University Medical Center Ho Chi Minh City, Ho Chi Minh City, Vietnam; ^3^ Department of Internal Medicine, University of Medicine and Pharmacy at Ho Chi Minh City, Ho Chi Minh City, Vietnam; ^4^ GI Endoscopy Department, University Medical Center Ho Chi Minh City, Ho Chi Minh City, Vietnam

**Keywords:** bile duct injuries, Strasberg classification, ERCP, laparoscopic cholecystectomy, hepaticocutaneous jejunostomy, Vietnam

## Abstract

**Objectives:**

To evaluate the clinical presentation, management, and outcomes of bile duct injuries (BDIs) after laparoscopic cholecystectomy (LC).

**Methods:**

This is a case series of 28 patients with BDIs after LC treated at a tertiary hospital in Vietnam during the 2006-2021 period. The BDI's clinical presentations, Strasberg classification types, management methods, and outcomes were reported.

**Results:**

BDIs were diagnosed intraoperatively in 3 (10.7%) patients and postoperatively in 25 (89.3%). The BDI types included Strasberg A (13, 46.4%), D (1, 3.6%), E1 (1, 3.6%), E2 (4, 14.3%), E3 (5, 17.9%), D + E2 (2, 7.1%), and nonclassified (2, 7.1%). Of the postoperative BDIs, the injury manifested as biliary obstruction (18, 72.0%), bile leak (5, 20.0%), and mixed scenarios (2, 8.0%). Regarding diagnostic methods, endoscopic retrograde cholangiopancreatography (ERCP) was more useful in bile leak scenarios, while multislice computed tomography, magnetic resonance cholangiopancreatography, and percutaneous transhepatic cholangiography were more useful in biliary obstruction scenarios. All 28 BDIs were successfully treated. ERCP with stenting was very effective in the majority of Strasberg A BDIs. For more complex BDI types, hepaticocutaneous jejunostomy was a safe and effective approach. The in-hospital morbidities included postoperative pneumonia (2, 10.7%) and biliary-enteric anastomosis leakage (1, 5.4%). There was no cholangitis or anastomotic stenosis during the follow-up after discharge (median 18 months).

**Conclusions:**

The majority of BDIs are type A and diagnosed postoperatively. ERCP is effective for the majority of Strasberg A BDIs. For major and complex BDIs, hepaticocutaneous jejunostomy is a safe and effective approach.

## Introduction

Laparoscopic cholecystectomy (LC) for symptomatic and complicated gallstone treatment is one of the most performed surgeries worldwide. Despite great benefits, such as minor postoperative pain and early recovery, LC has some complications, and one of the most severe complications is bile duct injury (BDI) (1, 2). Halbert and Fong et al. reviewed 850,000 LCs performed between 2005 and 2014 and reported an overall incidence of BDIs between 0.1% and 0.2% in the United States (3, 4). In Vietnam, LC was first performed in 1992 and has become a daily procedure in most hospitals. Nguyen et al. reported an incidence of BDIs of 0.92% among 1,028 LCs during the 1992–1998 period (5).

The diagnosis of BDI can be challenging. Owing to the development of endoscopy, minor BDIs can be effectively treated with stent placement via endoscopic retrograde cholangiopancreatography (ERCP). However, major and more complex BDIs require surgical intervention (6, 7). In addition to early postoperative complications, patients are also at high risk of biliary-enteric anastomotic strictures and recurrent episodes of cholangitis, which adversely affect their quality of life (8, 9). In Vietnam, only a few reports of BDIs after LC have been published (10). This study aimed to evaluate the clinical presentation, management, and outcomes of BDIs after LC at the University Medical Center (UMC) Ho Chi Minh City, Ho Chi Minh City, Vietnam.

## Materials and methods

This is a case series on BDI patients who were managed at UMC, one of the largest hepatobiliary centers in southern Vietnam, with approximately 1,300 LCs performed annually from May 2006 to May 2021. The study was approved by the Ethics Committee in Biomedical Research—University Medical Center Ho Chi Minh City (numbered 52/GCN-HDDD, signed on July 21, 2022). The management strategy for BDIs in our center during this period is as follows.

### Diagnosis approach for BDI

For BDIs that occur intraoperatively, the diagnosis is based on bile leakage from the tubular structure and confirmed by intraoperative cholangiography (IOC). For postoperative BDIs, abdominal pain, fever, jaundice, or bile leak from the abdominal drain should be the findings suspected of BDIs. Complete blood count, CRP, bilirubinemia, AST, ALT, abdominal ultrasonography, and multislice computed tomography (MSCT) scans should be performed. If a definite diagnosis cannot be made, magnetic resonance cholangiopancreatography (MRCP) should be performed. If MRCP was unavailable, percutaneous transhepatic cholangiography (PTC) was an alternative for biliary obstruction. In case the MSCT scan and MRCP results were inconclusive for bile leak, ERCP was performed.

**Figure 1:**
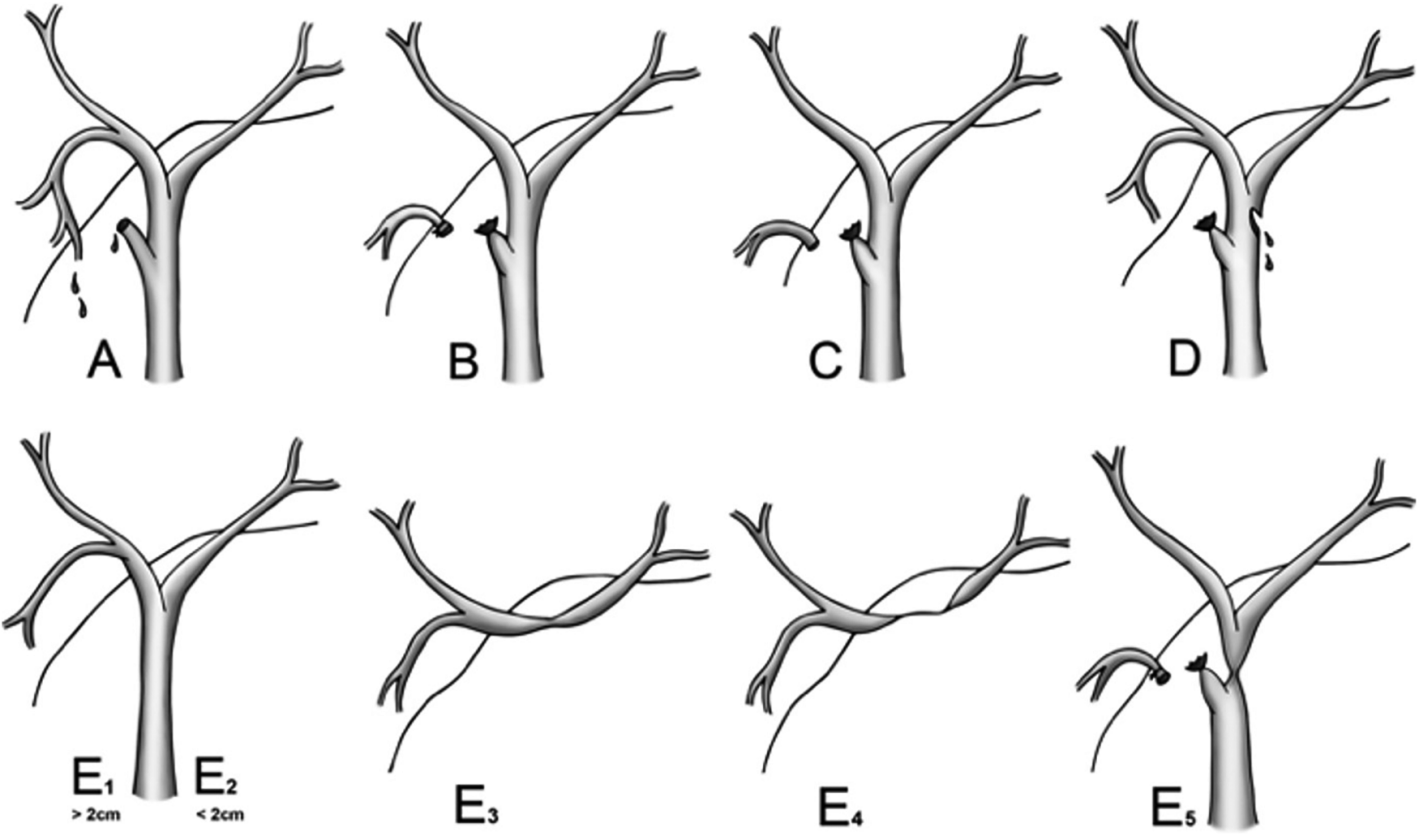
Strasberg classification of bile duct injury. Reproduced with permission from (11) Mbarushimana et al. © 2014 BMJ Publishing Group Ltd. All rights reserved.

BDIs were identified according to bile leakage, bile on abdominal paracentesis, or findings on MSCT, MRCP, ERCP, PTC, and surgery. The types of BDI were categorized according to the Strasberg classification (Figure 1) (11).

### Treatment strategy for BDI

The management includes conservative treatment, ERCP with stent placement, and surgery, depending on the time of diagnosis (intraoperative or postoperative), the type of BDI, and the patient's condition:

- Intraoperatively diagnosed BDIs: The injured bile duct could be sutured with or without choledochotomy, T-tube drainage, or hepaticocutaneous jejunostomy.
- Postoperatively diagnosed BDIs with bile leak scenario: We performed ERCP and stent placement for minor leakage without peritonitis. Conservative treatment was undertaken if ERCP failed and bile leakage was well drained. If the patient had postoperative local ascites, we performed ultrasound-guided percutaneous drainage. For patients with peritonitis, laparoscopic lavage, and injured bile duct suturing were performed if feasible. For Strasberg C injury, abdominal drainage followed by delayed hepaticocutaneous jejunostomy was performed. We sutured the CBD with T-tube drainage for Strasberg E injury if the common bile duct (CBD) was partially resected at less than 180^°^ in circumference. If the CBD resection was larger than 180^°^ in circumference, we performed hepaticocutaneous jejunostomy.
- Biliary obstruction scenario: For Strasberg B injury, percutaneous transhepatic biliary drainage was performed for biliary obstruction and cholangitis resolution. After that, hepaticocutaneous jejunostomy could be performed if the liver was not atrophied or atrophic liver was resected. For Strasberg E injury with complete CBD stenosis, we performed hepaticocutaneous jejunostomy. ERCP with stent placement or percutaneous transhepatic biliary stenting was performed for incomplete stenosis of the CBD and common hepatic ducts.

### Follow-up

For inpatients, daily careful clinical examination was undertaken. Blood tests (mainly complete blood count, CRP, bilirubin, AST, ALT) and abdominal ultrasonography were performed every three days. If postoperative surgical complications were suspected, we performed an MSCT scan. Antibiotics were used at least five days postoperatively. The patients were discharged six days after the surgery or stayed longer depending on the individual's condition.

For outpatients, clinical examination was undertaken every month for the first three months, every three months for the first year, and then annually. Blood tests and ultrasonography were utilized to surveil cholangitis and biliary-enteric anastomotic strictures. MSCT scans or MRCP were indicated if patients were suspected of cholangitis or biliary-enteric anastomotic stricture. For cholangitis diagnosis, we applied the diagnostic criteria and severity assessment according to the 2007 Tokyo Guidelines before 2013 (12). Since 2013, the Tokyo Guidelines 2013/2017 have been applied (13, 14). For biliary-enteric anastomotic stricture diagnosis, stricture was defined as the presence of bile duct dilatation and evidence of obstruction in MSCT or MRCP (15, 16).

The management was considered successful if the patient no longer had biliary leakage or biliary obstruction.

### Statistical analysis

All statistical analyses were carried out with SPSS 22.0 (SPSS Inc., Chicago, IL). Categorical data are presented as numbers and percentages. Quantitative data were tested for normality using the Kolmogorov–Smirnov test. Those with a normal distribution are presented as the mean and standard deviation, and those with a nonnormal distribution are presented as the median and interquartile range (IQR).

## Results

From May 2006 to May 2021, we had 28 patients diagnosed with BDIs in UMC. The mean age was 50 years (range, 14–89 years), and the male-to-female ratio was 1.3:1. Of the 28 BDIs, 8 (28.6%) were due to LC in our center, and 20 (71.4%) were transferred from other hospitals to our center. Three BDIs were diagnosed intraoperatively, and 25 BDIs were diagnosed postoperatively. The mean time from LC to the diagnosis of BDI was 26.7 days (ranging from 0 to 300 days).

### The manifestations and definite diagnosis of BDIs

The clinical manifestations of postoperative BDIs are shown in Table 1. The most common manifestations of bile leak and biliary obstruction were abdominal pain and jaundice, respectively. In 18 cases of bile leak, there were 9 from the abdominal drain, 3 peritonitis, 1 subdiaphragmatic fluid collection, and 5 ascites without peritonitis. All 5 cases of biliary obstruction presented with jaundice. In 2 cases of the combined scenario, 1 had a bile leak from the abdominal drain along with jaundice, and 1 had peritonitis with jaundice. There were two difficult diagnostic cases: one was initially misdiagnosed as cirrhosis due to abdominal fluid without abdominal pain and then revealed to be a bile leak owing to 800 ml of bile fluid via abdominal paracentesis. The other patient had abdominal pain, misdiagnosed as acute appendicitis, and surgical findings revealed BDI.

**Table 1:** Clinical manifestations of postoperative BDIs.

Clinical manifestation^a^	Bile leak scenario *n* = 18 (%)	Biliary obstruction scenario *n* = 5 (%)	Combined scenario *n* = 2 (%)	Total *n* = 25 (%)
Bile leakage, *n (%)*	9 (50)	0 (0)	1 (50.0)	10 (40.0)
Jaundice, *n (%)*	5 (27.8)	5 (100)	2 (100)	12 (48.0)
Ascites, *n (%)*	2 (11.1)	0 (0)	0 (0)	2 (8.0)
Abdominal pain, *n (%)*	13 (72.2)	2 (40.0)	2 (100)	17 (68.0)
Fever, *n (%)*	4 (22.2)	3 (60.0)	1 (50.0)	8 (32.0)

^a^ One scenario may have one or more symptoms. The percentage in parentheses is calculated by the number of cases with that symptom divided by the total number of cases of the corresponding scenario.

**Figure 2:**
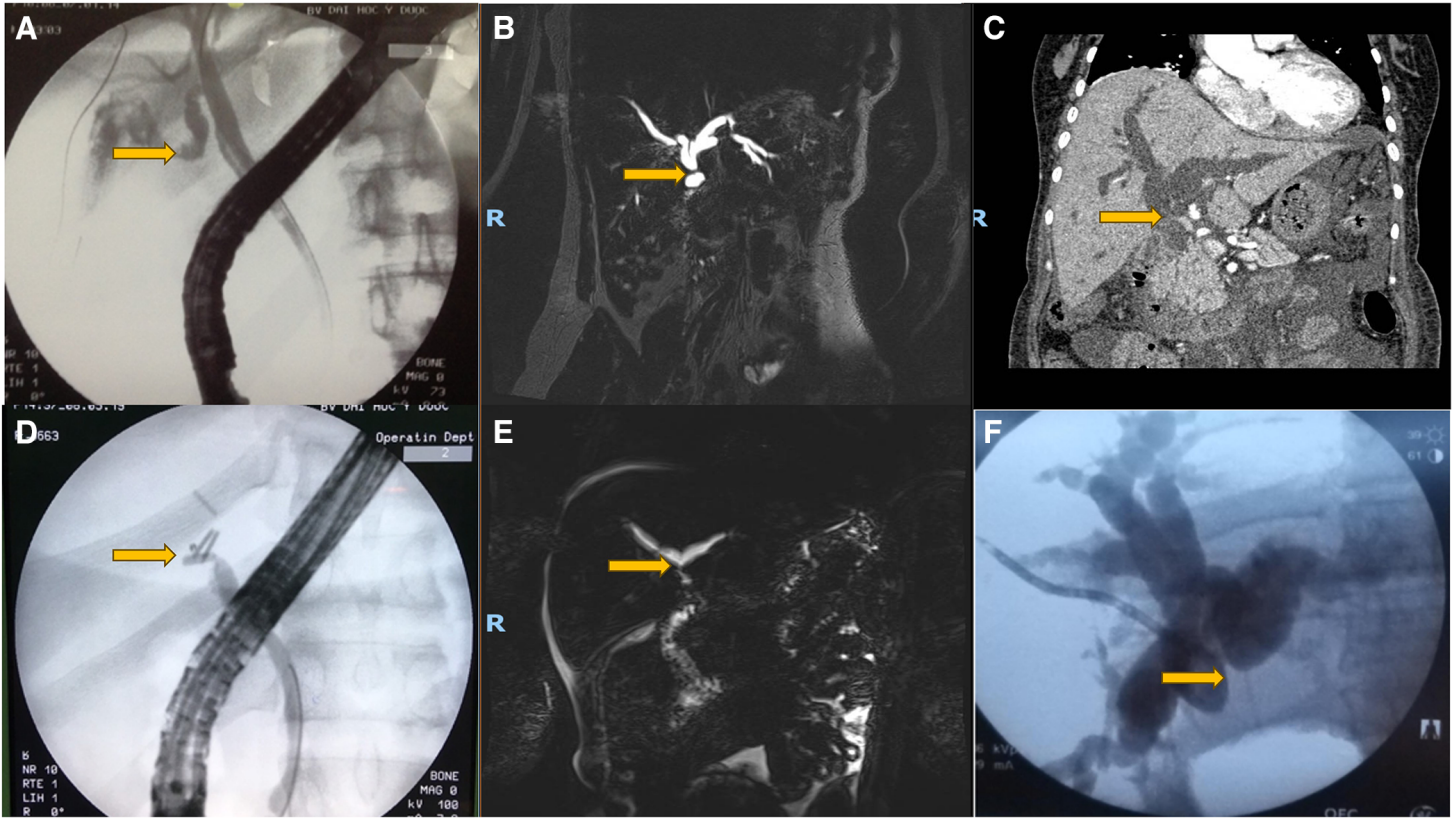
BDIs in different imaging modalities. (**A**) Strasberg a injury—fistula from the cystic duct (arrow) on ERCP. (**B**) Strasberg E2 injury (arrow) on MRCP. (**C**) Strasberg E2 injury (arrow) on MSCT scan. (**D**) Abrupt cutoff CBD due to surgical clip (arrow) on ERCP. (**E**) Strasberg E3 injury (arrow) on MRCP. (**F**) Strasberg E3 injury (arrow) on PTC.

For definite BDI diagnosis, we applied MSCT scan, MRCP, ERCP, and PTC, as shown in Table 2. Only 1 BDI (10%) was definitively diagnosed for the MSCT scan. This patient had a biliary obstruction scenario and was finally revealed to have an E2 injury (Figure 2C). The other nondiagnostic BDIs were classified owing to ERCP in 4, PTC in 1, and surgery in 3, and the remaining case could not be classified due to undergoing conservative treatment without further investigations. When using MRCP, only 3 BDIs (27.3%) were definitively diagnosed: 1 of a bile leak scenario and 2 of a combined scenario (Figure 2B–E). One was classified as a Strasberg E2 injury on MRCP, and the surgical findings revealed another missed Strasberg D injury. The remaining nondiagnostic BDIs were then diagnosed due to ERCP in 4 patients and surgery in the other 4 patients. Of the 18 patients who underwent ERCP, 17 (94.4%) were successfully treated. In successful cases, 10 BDIs (58.8%) with bile leak presentation were diagnosed (Figure 2A); 6 BDIs (35.3%) could not be clarified with images of abrupt CBD cutoff on ERCP (Figure 2D); 1 Strasberg A BDI (5,9%), confirmed by ERCP with bile leak from the cystic duct, accidentally was found another bile leak from the Luschka duct that missed on ERCP. Nondiagnostic BDIs on ERCP were then classified based on MSCT scan in 2, MRCP in 1, PTC in 1, and surgery in 4. PTC was utilized to classify BDIs in 2 biliary obstructive cases, which could not be confirmed on MSCT scan and ERCP (Figure 2F). These patients had obstructive cholangitis and required percutaneous transhepatic biliary drainage.

**Table 2:** Diagnosis methods for postoperative BDIs.

	Definitive diagnosis of BDI	Bile leak scenario *n* = 18	Biliary obstruction scenario, *n* = 5	Combined scenario *n* = 2	Total *n* = 25
MSCT Scan *n* = 10	Yes	0	1	0	1
	No	8	1	0	9
MRCP *n* = 11	Yes	0	1	2	3
	No	8	0	0	8
ERCP *n* = 18	Yes	10	0	0	10
	No	3	3	1	7
	Fail	1	0	0	1
PTC *n* = 2	Yes	0	2	0	2
	No	0	0	0	0

MSCT, multislice computed tomography; MRCP, magnetic resonance cholangiopancreatography; ERCP, endoscopic retrograde cholangiopancreatography; PTC, percutaneous transhepatic cholangiography.

When preoperative imaging modalities could not make a definite diagnosis, the surgical findings played a crucial role in identifying the lesions. Among 11 surgically identified BDIs, one was intraoperatively found to have the right hepatic artery clipped. In this patient, liver function was not affected, and the liver was found to have no ischemia intraoperatively. Therefore, we did nothing to the clipped right hepatic artery. Finally, we had two BDIs that could not be identified. These patients had bile leakage through the abdominal drainage tube. Abdominal ultrasounds found fluid collection in the gallbladder bed, and both underwent successful conservative treatment. The types of BDI in our study according to the Strasberg classification are summarized in Table 3.

**Table 3:** BDI type according to the Strasberg classification and diagnostic methods.

Diagnosis methods	BDI type according to the Strasberg classification							Total *n *= 28
	A	D	E1	E2	E3	D + E2	Nonclassified	
Clinical finding, *n* (%)	–	–	–	–	–	–	2 (7.1)	2 (7.1)
MSCT Scan *n* (%)	–	–	–	1 (3.6)	–	–	–	1 (3.6)
MRCP, *n* (%)	–	–	–	1 (3.6)	1 (3.6)	–	–	2 (7.1)
ERCP, *n* (%)	10 (35.8)	–	–	–	–	–	–	10 (35.8)
PTC, *n* (%)	–	–	–	–	2 (7.1)	–	–	2 (7.1)
Surgery, *n* (%)	3 (10.7)	1 (3.6)	1 (3.6)	2 (7.1)	2 (7.1)	2 (7.2)	–	11 (39.3)

MSCT, multislice computed tomography; MRCP, magnetic resonance cholangiopancreatography; ERCP, endoscopic retrograde cholangiopancreatography; PTC, percutaneous transhepatic cholangiography.

### Management and outcomes

The treatment methods are shown in Table 4. The mean interval for BDI management was 37.6 days, ranging from 0 to 300 days. There was one bile leak from the cystic duct (Strasberg A), which was merely laparoscopically sutured. The patient then had a recurrent bile leak and developed a subdiaphragmatic abscess. We had to reoperate laparoscopically to suture the fistula and perform choledochotomy followed by T-tube drainage.

**Table 4:** Treatment methods according to the type of BDI.

Treatment methods		Strasberg BDIs					Total (%) *n *= 28
		A *n *= 13	D *n *= 1	E1, E2, E3 *n *= 10	D + E2 *n *= 2	Nonclassified *n *= 2	
Conservative treatment		–	–	–	–	2 (7.1)	2 (7.1)
ERCP and stent placement	Only stenting	6 (21.4)	–	–	–	–	6 (21.4)
	Combined biloma drainage	1 (3.6)	–	–	–	–	1 (3.6)
	Combined laparoscopic lavage	3 (10.7)	–	–	–	–	3 (10.7)
	Combined laparoscopic lavage and accessory bile duct suturing	1 (3.6)	–	–	–	–	1 (3.6)
Laparoscopic cystic duct ligating and abdominal lavage		1 (3.6)	–	–	–	–	1 (3.6)
Laparoscopic CBD suturing and T-tube drainage		1 (3.6)	1 (3.6)	–	–	–	2 (7.1)
Hepaticocutaneous jejunostomy		–	–	10 (35.8)	2 (7.1)	–	12 (42.9)

ERCP, Endoscopic retrograde cholangiopancreatography; CBD, common bile duct.

For short-term outcomes (Table 5), the early morbidity was 10.7%. 2 cases of postoperative pneumonia were successfully managed and discharged, and 1 case of biliary-enteric fistula was successfully managed with conservative treatment. The mortality was 0%.

**Table 5:** Short-term outcomes of patients in this study.

Treatment methods		Successful (%)	Fail (%)	The mean length of hospital stay (days)	Early complications after intervention (%)	Death (%)
Conservation treatment, *n* = 2		2 (100)	0	11.5 (10–13)	0	0
ERCP and stent placement *n* = 12	Only stenting *n* = 7	11 (91.7)	1 (8.3)	7.3 (3–14)	0	0
	Combined biloma drainage *n* = 1					
	Combined laparoscopic lavage *n* = 3					
	Combined laparoscopic lavage and accessory bile duct suturing *n* = 1					
Laparoscopic cystic duct ligating and abdominal lavage, *n* = 1		1 (100)	0	7	0	0
Laparoscopic CBD suturing and T-tube drainage, *n* = 2		2 (100)	0	15.5 (12–19)	1 (3.6)	0
Hepaticocutaneous jejunostomy *n* = 12		12 (100)	0	11.1 (5–25)	2 (7.1)	0

Five cases, including two with conservative treatment, one with ERCP and stent placement, and two with hepaticocutaneous jejunostomy, could not be followed up for long-term outcomes (Table 6). The remaining 23 patients had a follow-up period from 1 to 144 months. One (3.6%) case of acute cholangitis was due to stent occlusion, which occurred two months after ERCP and stent placement. The stent was then removed. There was no bile leak or bile duct stricture.

**Table 6:** Long-term outcomes of patients in this study.

Treatment methods		Follow-up (months)	Cholangitis (%)	Bile duct stricture (%)
Conservation treatment, *n* = 2		Lost	Lost	Lost
ERCP and stent placement *n* = 11	Only stenting, *n* = 6	19.5 (1–144)	1 (9.1)	0
	Combined biloma drainage, *n* = 1			
	Combined laparoscopic lavage, *n* = 3			
	Combined laparoscopic lavage and accessory bile duct suturing, *n* = 1			
Laparoscopic cystic duct ligating and abdominal lavage, *n* = 1		4	0	0
Laparoscopic CBD suturing and T-tube drainage, *n* = 2		2.5 (2–3)	0	0
Hepaticocutaneous jejunostomy, *n* = 12		26.2 (1–82)	0	0

## Discussion

Cholecystectomy is one of the most common abdominal surgical procedures. In the United States, 90 percent of cholecystectomies are performed laparoscopically (17). LC is considered the “gold standard” for the surgical management of gallstone disease. The procedure results in less postoperative pain, better cosmesis, shorter hospital stays, and less disability from work than open cholecystectomy (18, 19). In Vietnam, LC has been performed for nearly three decades and has become a popular procedure for the management of symptomatic and complicated gallstone disease. If patients had symptomatic gallstones, LC was the first choice. If patients had gallstone-induced cholecystitis, LC or a two-stage surgical plan (i.e., percutaneous transhepatic gallbladder drainage in the first stage and then LC in the second stage) was considered depending on the patient's condition, as guided by the 2018 Tokyo guidelines (20).

BDI is a severe complication of LC that adversely affects the patient's quality of life (1, 2). In Vietnam, this complication tends to occur in difficult cholecystectomies or in the hands of junior surgeons. The management of BDI is mainly based on the timing of the BDI diagnosis, the locally available devices, and the surgeon's experience. If the surgeon is not a hepatobiliary specialist or the necessary devices are not available, drains will be placed, and patients will be transferred to tertiary centers, including ours, for definite repair. This approach is the same as that in other centers around the world (21, 22).

In our study, the most common type of BDI after LC was bile leak from the cystic duct (Strasberg A), accounting for 46.4% of cases. Similarly, Viste A. and Arcerito M. reported that Strasberg A accounted for 52.2% and 44.2% of all BDIs, respectively (23, 24). BDI may present as bile leak or biliary obstruction or in a combined scenario. It can be easily diagnosed when typical findings are presented, such as bile leakage via abdominal drainage tubes, biliary peritonitis, or obstructive jaundice after LC. We had 2 cases with difficult diagnoses: one was initially misdiagnosed as cirrhosis, and the other was misdiagnosed as appendicitis. Both had moderate abdominal fluid but vague abdominal pain. In the former patient, abdominal paracentesis was performed, which confirmed bile leak. Therefore, we suggest that all patients with free abdominal fluid after LC should be suspected of bile leak until proven otherwise. In some situations where imaging is not diagnostic, abdominal paracentesis can help confirm the diagnosis.

The imaging modalities played different roles in BDI identification. In the biliary obstruction scenario, MSCT scans could help classify BDIs. However, the image was sometimes unclear, making it challenging to identify the exact location of the bile duct stricture. In addition, MSCT could not help identify the bile leak location in the bile leak scenario. Gorsi U., et al. reported similar results (25). MRCP was valuable in classifying biliary obstructive lesions but could not identify the bile leak location, similar to the MSCT scan. According to Kantarci et al., the sensitivity, specificity, and accuracy of MRCP in BDI diagnosis were 63.6%, 51.8% and 57.1%, respectively (26). According to other authors, MRCP only identifies fluid accumulation or ascites. However, there was no direct image of the bile leak location. Therefore, it was challenging to distinguish BDIs from other abnormalities (25–29). ERCP was valuable not only for diagnosis but also for the management of BDI. In our study, ERCP was able to identify a bile leak from the cystic duct but could miss the bile leak from the accessory bile duct as well as unable to identify the CBD cutoff lesions. These results were similar to those of previous studies (30, 31). We had 2 BDI cases with biliary obstruction, which were finally confirmed to be E3 type by PTC (due to unclassifiable MSCT scan and ERCP findings). We only performed PTC for obstructive cholangitis. In cases with bile leak, the bile ducts were not dilated, and it was very challenging to perform this procedure. Our findings were also consistent with other studies (32, 33). From the above results, ERCP is the first choice for BDIs with bile leak, and MRCP is the first choice for BDIs with biliary obstruction. PTC is a suitable alternative when MRCP is unavailable or the patient needs biliary drainage due to cholangitis.

Regarding management methods, most authors believed that simple BDIs could be successfully managed with ERCP and stent placement, while complicated BDIs require surgical intervention (6, 7). Our results were similar to those of other studies. For minor bile leaks, we performed ERCP and stent placement. If the patients had localized intraabdominal fluid accumulation, we performed ERCP and stent placement in combination with ultrasound-guided percutaneous drainage. If the patients had biliary peritonitis or biliary ascites, we performed ERCP for stent placement and laparoscopic lavage and drainage. Our early results were similar to previous reports (Table 7). After follow-up from 1 to 144 months, there was 1 (9.1%) case of acute cholangitis due to stent obstruction.

**Table 7:** Early results of ERCP and stent placement for BDIs.

Study	Year	Country	*N*	Success (%)	Morbidity (%)
Familiari et al. (34)	2003	Italy	85	96.3	2.9
Mavrogiannis et al. (35)	2006	Greece	52	100	7.7
Katsinelos et al. (36)	2008	Greece	60	94.0	13.0
Donnellan et al. (37)	2009	Ireland	48	91.7	0
Tzovaras et al. (38)	2009	Greece	20	95.0	5.2
Hii et al. (39)	2011	Australia	29	93.1	6.9
Arcerito et al. (23)	2019	USA	23	100	0
Our study	2023	Vietnam	12	91.7	0

When ERCP failed or was not available, we performed laparoscopic surgery. We had one patient with Strasberg D. We sutured the fistula and performed choledochotomy and T-tube insertion. The patient had no recurrent bile leak. We found that biliary drainage was essential for fistula healing and reducing the risk of recurrent bile leak in BDIs from the CBD. This finding was similar to that in the study of Hii M. et al, which showed that bile duct drainage played a crucial role in fistula healing for Strasberg A BDI (39). There was another bile leak from the cystic duct. The leakage location was adjacent to the confluence of the cystic duct to the CBD. The patient underwent laparoscopic surgery for suturing of the fistula by nonbiliary surgeons. However, he had a recurrent bile leak and a subdiaphragmatic abscess. We reoperated laparoscopically to suture the fistula and performed choledochotomy followed by T-tube drainage. The patient no longer had bile leak. Abdel Rafee A. et al. and Stewart L. et al. recommended that BDI should be repaired by experienced hepatobiliary surgeons for optimal outcomes (21, 22).

For Strasberg E, we performed hepaticocutaneous jejunostomy surgery. The morbidity rate in our study was 7.1%. Goméz D. et al. (40) reported an overall complication rate of 10%. One patient in their study presented bile leak (type E4), and another patient required admission to the intensive care unit after reintervention. Ahmad H. et al. (41) reported 87 BDIs that underwent hepaticojejunostomy with an overall morbidity rate of 51.7%. Five (5.7%) patients had posthepaticojejunostomy bile leak. Conservative management was performed in four (4.5%) patients, and laparotomy for bile leak from a duodenal injury was required in one patient. The mortality was 2.3%, and both were due to septic shock. In our study, hepaticocutaneous jejunostomy was preferred to hepaticojejunostomy for some reasons. With this technique, we usually placed transanastomotic biliary drains unilaterally or bilaterally via the jejunal stump to check for anastomotic leakage intraoperatively and to protect and improve the patency of the anastomosis postoperatively. We had one patient suffering from biliary-enteric fistula with low biliary output via abdominal drain. The biliary output had reduced day after day for nearly one month without any further intervention. We supposed that this patient had successful conservative management thanks to transanastomotic biliary drain. Most patients with hepaticocutaneous jejunostomy underwent cholangiography three months postoperatively. If the cholangiogram found neither anastomotic leakage nor stenosis, we removed the transanastomotic biliary drain. In our study, neither anastomotic leakage nor stenosis was documented after three months.

The optimal timing for the repair of severe BDIs with hepaticocutaneous jejunostomy is an important issue. In our study, the timing of the repair was up to individuals, based on the overall patient's condition, concomitant morbidities, and the condition of abdominal cavity infection (i.e., with or without peritonitis). This finding was in accordance with a previous study of the European-African HepatoPancreatoBiliary Association (E-AHPBA) (42). In this retrospective multicenter study, patients who underwent hepaticojejunostomy after BDI from January 2000 to June 2016 were classified according to the timing of biliary reconstruction with hepaticojejunostomy: early (days 0–7), intermediate (1–6 weeks) and late (6 weeks–6 months). The authors concluded that the timing of biliary reconstruction with hepaticojejunostomy did not have any impact on severe postoperative complications, the need for reintervention, or liver-related mortality, and individualized treatment after iatrogenic BDI was still advisable (42).

Our long-term outcomes were comparable to those of other authors, as summarized in Table 8. The risk factors for biliary-enteric anastomotic stricture have been reported in several previous studies. According to Viste et al. (24), there might be several reasons for developing strictures, such as technical failures during reconstruction, unawareness of constrained blood supply, or extensive damage making anastomoses difficult. Hajjar et al. (43) stated that postoperative biliary leakage was a significant independent predictive factor for late anastomotic stricture. Stewart et al. (22) reported that intra-abdominal infection, surgical technique, and surgeon experience were risk factors for biliary-enteric anastomotic stricture. In AbdelRafee's study (21), post-ERCP pancreatitis and postoperative bile leak were independent risk factors for poor outcomes. Walsh et al. (9) concluded that the level of injury and the timing of repair helped to predict the risk of postoperative stricture. We had 2 cases with high-risk factors for biliary-enteric anastomotic stricture. One case of biliary-enteric anastomotic leakage had no biliary-enteric anastomotic stenosis after 58 months of follow-up. Another case had a clipped right hepatic artery, which showed no narrowing of the biliary-enteric anastomosis after 14 months. These factors still have controversial effects on the risk of anastomotic stricture. While some studies have shown similar long-term outcomes (44), several other studies have demonstrated an increased risk of postoperative bile duct strictures in patients with combined bile and vascular injuries (45, 46). Our current results are quite good, but the follow-up duration should be extended, as most of the cases have been followed for less than five years. In addition, the number of patients in our study may not be large enough to evaluate all long-term complications. Most previous authors recommended a minimum follow-up period of 5 years (47), and some even suggested follow-up of patients up to 10–20 years (48). AbdelRafee argued that even after 20 years, patients were still at risk of biliary-enteric anastomotic stricture (21).

**Table 8:** Long-term outcome after hepaticocutaneous jejunostomy surgery.

Study	Year	Country	*N*	Follow-up period (months)	Biliary-enteric anastomotic stricture rate (%)	Cholangitis rate (%)
Walsh et al. (9)	2007	USA	84	67	11.0	6.0
Hajjar et al. (43)	2014	Romania	36	12–68	11.7	NA
Viste et al. (24)	2015	Norway	22	NA	13.6	NA
AbdelRafee et al. (21)	2015	Egypt	120	180	11.6	14.2
Goméz et al. (40)	2020	Colombia	20	12–60	0	0
Ahmad et al. (41)	2023	Pakistan	87	2–74	5.7	0
Our study	2023	Vietnam	12	1–82	0	0

NA, not available.

In addition to the results that were almost similar to those of previous studies, we reported on the hepaticocutaneous jejunostomy method, which was routinely applied in complicated BDIs in our study. We found that this technique might help protect the biliary-enteric anastomosis. If patients had anastomotic leakage, it would increase the likelihood of successful conservative treatment. If patients had anastomotic stenosis, a subcutaneous intervention technique such as dilating or stenting might be utilized via a subcutaneous jejunal tunnel.

Our study had some limitations. First, this is a case series with a limited number of patients. Second, some data might have been missed due to the retrospective nature of this study. Third, the results of this study might have limited generalizability, as the outcomes were based on data from a single center.

## Conclusions

The most common type of BDI after LC is Strasberg A. BDIs can be easily diagnosed when patients have typical presentations, such as bile through an intraabdominal drainage tube, biliary peritonitis, or obstructive jaundice after LC. MSCT scan, MRCP, and PTC are valuable in making a definite diagnosis of BDIs causing biliary obstruction but have little value in diagnosing BDIs causing bile leakage. ERCP is a good method to identify the bile leak location (except for cutoff CBD), and it is an effective treatment for minor bile leak (i.e., Strasberg A). When ERCP fails, laparoscopic CBD suturing and T-tube drainage is the valuable alternative. For major BDIs, hepaticocutaneous jejunostomy is a safe and effective method with a low incidence of biliary-enteric anastomotic stricture and cholangitis. These results should be further evaluated by future studies with sufficiently large sample sizes and a minimum follow-up period of 5 years.

## Data Availability

The raw data supporting the conclusions of this article will be made available by the authors, without undue reservation.
